# Dynamic Contrast Enhanced MRI Detects Early Response to Adoptive NK Cellular Immunotherapy Targeting the NG2 Proteoglycan in a Rat Model of Glioblastoma

**DOI:** 10.1371/journal.pone.0108414

**Published:** 2014-09-30

**Authors:** Cecilie Brekke Rygh, Jian Wang, Marte Thuen, Andrea Gras Navarro, Else Marie Huuse, Frits Thorsen, Aurelie Poli, Jacques Zimmer, Olav Haraldseth, Stein Atle Lie, Per Øyvind Enger, Martha Chekenya

**Affiliations:** 1 Department of Biomedicine, University of Bergen, Bergen, Norway; 2 Cardiovascular Research Group, Haukeland University Hospital, Bergen, Norway; 3 MI Lab, Department of Circulation and Medical Imaging, NTNU, Trondheim, Norway; 4 Molecular Imaging Center, Department of Biomedicine, University of Bergen, Bergen, Norway; 5 Laboratoire d'Immunogénétique-Allergologie, CRP-Santé, Luxembourg City, Luxembourg; 6 Department of Medical Imaging, St. Olavs Hospital, Trondheim, Norway; 7 Institute for Clinical Dentistry, University of Bergen, Bergen, Norway; 8 Department of Neurosurgery, Haukeland University Hospital, Bergen, Norway; Cedars-Sinai Medical Center, United States of America

## Abstract

There are currently no established radiological parameters that predict response to immunotherapy. We hypothesised that multiparametric, longitudinal magnetic resonance imaging (MRI) of physiological parameters and pharmacokinetic models might detect early biological responses to immunotherapy for glioblastoma targeting NG2/*CSPG4* with mAb9.2.27 combined with natural killer (NK) cells. Contrast enhanced conventional T1-weighted MRI at 7±1 and 17±2 days post-treatment failed to detect differences in tumour size between the treatment groups, whereas, follow-up scans at 3 months demonstrated diminished signal intensity and tumour volume in the surviving NK+mAb9.2.27 treated animals. Notably, interstitial volume fraction (v_e_), was significantly increased in the NK+mAb9.2.27 combination therapy group compared mAb9.2.27 and NK cell monotherapy groups (p = 0.002 and p = 0.017 respectively) in cohort 1 animals treated with 1 million NK cells. v_e_ was reproducibly increased in the combination NK+mAb9.2.27 compared to NK cell monotherapy in cohort 2 treated with increased dose of 2 million NK cells (p<0.0001), indicating greater cell death induced by NK+mAb9.2.27 treatment. The interstitial volume fraction in the NK monotherapy group was significantly reduced compared to mAb9.2.27 monotherapy (p<0.0001) and untreated controls (p = 0.014) in the cohort 2 animals. NK cells in monotherapy were unable to kill the U87MG cells that highly expressed class I human leucocyte antigens, and diminished stress ligands for activating receptors. A significant association between apparent diffusion coefficient (ADC) of water and v_e_ in combination NK+mAb9.2.27 and NK monotherapy treated tumours was evident, where increased ADC corresponded to reduced v_e_ in both cases. Collectively, these data support histological measures at end-stage demonstrating diminished tumour cell proliferation and pronounced apoptosis in the NK+mAb9.2.27 treated tumours compared to the other groups. In conclusion, v_e_ was the most reliable radiological parameter for detecting response to intralesional NK cellular therapy.

## Introduction

Glioblastoma (GBM) is a highly aggressive brain tumour where the patients' median survival is only 14.6 months [1] despite aggressive multimodal treatment comprising debulking surgery, temozolomide (TMZ) given concurrently with fractionated radiotherapy and additional adjuvant TMZ [1]. This dismal prognosis is partly due to the diffuse infiltrative nature of GBMs that invariably results in recurrence within 2 cm of the original surgical margin [2]. Their molecular heterogeneity, the variable disruption of the blood brain barrier (BBB) and high tumour interstitial pressure [3] renders GBM therapy resistant and hinders the entry of cytotoxic agents, including lymphocytes into the tumour. According to the imaging response criteria for high-grade gliomas, the RANO (Response Assessment in Neuro-Oncology) criteria [4], the treatment effect is evaluated based on changes in the solid tumour size (bi-dimensionally measured in contrast-enhancing lesions) and not the underlying pathophysiological changes that may precede changes in morphology. More specified criteria for evaluating glioblastoma in trials of anti-angiogenic therapy were recently proposed [5]. However, the main criteria are still based on the morphological changes detected by magnetic resonance imaging (MRI) or computer tomography (CT). Since development of local therapies that target the aggressive cell types within GBMs is the benchmark, imaging modalities that can confirm early treatment efficacy in the tumour bed are highly needed.

We previously demonstrated that GBMs express high levels of Neuron-glia 2 (NG2), a cell surface chondroitin sulphate proteoglycan (CSPG4), that confers proliferative [6]–[8] and angiogenic potential [7], [9], [10] and mediates resistance to chemo- and radiotherapy [11], [12]. Consequently, high NG2 expression in GBM biopsies is prognostic for shorter patient survival [12]. NG2 is aberrantly expressed by several other tumour types [13]–[15] and has been shown to mediate their malignant progression [15]. As a cell surface molecule, with expression restricted to tumour cells, and mediating an aggressive disease course, NG2 may be a good target for immunotherapy. In a recent study, we targeted NG2/CSPG4 with monoclonal antibody 9.2.27 (mAb9.2.27) combined with *in vitro* activated natural killer cells (NK) in an intralesional adoptive cellular immunotherapy approach [16], [17]. We demonstrated that combination NK+mAb9.2.27 treatment converted the tumour promoting, anti-inflammatory microenvironment to a pro-inflammatory one mediated by M1-like macrophage/microglia. NK+mAb9.2.27 treatment diminished tumour growth and prolonged animal survival [16].

NK cells are large granular lymphocytes that are involved in both innate and adaptive immune responses and are highly cytotoxic against tumour and virus infected cells. Among cytotoxic lymphocytes, NK cells are the most efficient effectors against tumours, and are capable of direct killing without prior immunization [18]. In human, they recognise targets for killing through ligation of inhibitory killer immunoglobulin like receptors (KIRs) to class I human leucocyte antigens (HLA), and functionally similar systems exist in rats and mice. Ligation of inhibitory KIRs to their cognate class I HLA ligands, transduces an inhibitory signal that, in the absence of activating signals, renders NK cells hyporesponsive [19], [20]. NK cells are highly attractive for GBM treatment because they have been demonstrated to preferentially kill GBM stem-like cells [21], [22]. Moreover, interleukin-2 (IL-2) activated NK cells express high levels of CD16, a low affinity FcγRIII receptor that binds antibody Fc domains to mediate potent antibody dependent cellular cytotoxicity (ADCC) of coated target cells.

One of the greatest challenges in developing therapeutic regimens is the inability to rapidly and objectively assess the tumour's physiological responses to treatment *in situ*. Moreover, the tumour's response to therapy in many cases is transient. Therefore, quantitative measures to characterize cancer progression are required for differential diagnosis and therapeutic monitoring. Imaging techniques, such as MRI, permit diagnosis, non-invasive, longitudinal monitoring of progression and potential responses to anti-cancer treatment. Discrimination of the boundaries between malignant and normal tissue and the evaluation of cellular heterogeneity in lesions has proven to be a limitation in conventional MRI. The use of multiparametric, longitudinal MRI imaging and subsequent data analyses may provide more accurate information and hence, improved tumour lesion characterisation [9]. Thus, we aimed to prospectively identify quantifiable biological parameters by both morphological and physiological MRI that may reveal clinically relevant, tumour physiological changes that may be apparent before structural changes are evident. Dynamic contrast enhanced MRI (DCE MRI) is increasingly used in oncology to investigate aspects of tumour microcirculation and to quantify changes during treatment [23]. The Tofts' model [24] is a two-compartment pharmacokinetic model, which allows estimation of the transfer constant (K^trans^), extracellular extravascular volume fraction (v_e_) and blood plasma volume fraction (v_p_). Diffusion weighted MRI (DWI) detects changes in water mobility and has been applied to deduce the cellularity of the tumour and thus of treatment response [25], [26]. Herein, we combined DCE MRI and DWI with conventional morphological MR imaging such as T2-weighted, pre- and post-contrast T1-weighted imaging to identify imaging parameters that may be useful for detecting early signs of treatment effects. We hypothesized that early biological changes that correlate with response to treatment can be detected by physiological MRI before they are measurable by histology or in conventional MR images as altered tumour volumes. In order to monitor tumour progression longitudinally, post-treatment multiparametric MRI was performed at two time points. We also aimed to investigate whether the measured ADC values in the tumours associate with the estimated perfusion parameters such as the extracellular volume fraction v_e_ since both parameters may provide information about the tumour matrix and cell density. In this study, v_e_ proved to be the most reliable parameter for detection of early treatment effects.

## Materials and Methods

### Tumour cell culture and flow cytometry characterisation

The U87MG cell line was purchased from the American Type Culture Collection, (ATCC; Rockville, MA) and was propagated and fingerprinted as previously described [7], [12].

The cells were immunolabelled in a final volume of 100 µl of FACS buffer (Miltenyi Biotec, Scheelavägen, Sweden) with PCPCY5.5-conjugated anti-nestin (1∶20 dilution, 25/NESTIN clone, BD Biosciences, Trondheim, Norway), PE-conjugated anti-vimentin (1∶20, RV202, BD Biosciences), FITC-conjugated anti-GFAP (1∶25, GA5, eBioscience), ligands for NK cell receptors were detected using AlexaFluor488-conjugated anti-MICA (1∶40, 159227, R&D Systems, UK), APC-conjugated anti-MICB (1∶40, 236511, R&D Systems), unconjugated anti-ULBP-1 (1∶10, 170818, R&D Systems), unconjugated anti-ULBP-2-5-6 (1∶10, 166510, R&D Systems) and unconjugated anti-ULBP-3 (1∶10, 166510, R&D Systems) stained with Pacific Orange- conjugated Fragment of goat anti-mouse-IgG (1∶40, Invitrogen) and HLA molecules were immunolabelled with APC-conjugated anti-HLA-A,B,C (1∶5, G46-2,6, BD Biosciences), PE-conjugated anti-HLA-E (1∶20, 3D12HLA-E, eBioscience), PE-conjugated anti-HLA-G (1∶20, 87G, Biolegend, San Diego, CA) and FITC-conjugated anti-HLA-DR,DP,DQ (1∶5, Tu39, BD Biosciences). V450 Horizon-conjugated IgG1 (1∶20, MOPC-21, BD Biosciences), FITC-conjugated anti-IgG2a (1∶20, PPV-04, Immunotools, Friesoythe, Germany) and FITC-conjugated9 anti-IgG2b (1∶20, PLRV219, Immunotools) were used as isotype controls. All samples were stained with Live/Dead Fixable Near-IR Dead Cell Stain Kit (Invitrogen) in order to gate out dead cells. Data analysis was performed in FACSDiva Software (BD Biosciences).

### Animals

8–10 week old immunodeficient nude rats (Han: rnu/rnu Rowett Nude), of both sexes were included in the study. The athymic nude rat is deficient of some T-cell subtypes, but has normal complement and B-cell function [27]. The animals were bred in an isolation facility at 25°C (55% relative humidity), 12/12 hr light cycle, in a specific pathogen free environment and animal husbandry protocols were maintained as previously described [7]. All animal procedures were performed in accordance with protocols approved by The National Animal Research Authority (Oslo, Norway). The animals were inspected daily and were sacrificed by CO2 inhalation and decapitation when they developed neurological symptoms such as lethargy and/or paralysis, neglected grooming, rotational behaviour, and dome head. The brains were extracted for further analysis.

### Acute dissociation of splenocytes, NK cell purification and implantation

Rat NK cells were purified from spleens of littermates by negative selection and cultured for 4–5 days in the presence of 1000 U/ml human recombinant IL-2 (R&D Systems) as previously described [28]. Tumour spheroids (each containing 30,000 cells) [29] were selected under a stereo light microscope and 32 animals were xenografted with 15 U87MG spheroids. The animals were anaesthetized with subcutaneous injection of Hypnorm-Dormicum (0.4 ml/kg) – Roche, Indianapolis, USA), the head secured in a stereotactic frame (Benchmark; Neurolab, St Louis, MO) and a short longitudinal incision was made in the scalp exposing the calvarium. A burr-hole was made 1 mm posterior to the bregma and 3 mm right lateral to the sagittal suture with a micro-drill with a bit diameter of 2.9 mm. A Hamilton syringe with inner diameter of 810 µm was introduced to a depth of 2.5 mm below the brain surface, and the spheroids suspended in 10 µl of phosphate buffered saline (PBS) were slowly injected and the syringe left in place for 3 min before withdrawal. The skin was closed with an Ethilon 3–0 suture. We treated two cohorts of animals, and in both cohorts the tumours were allowed to establish for 3 weeks prior to random assignment to treatment with phosphate buffered saline (vehicle control), mAb9.2.27, NK cells (cohort 1: 1 million cells, cohort 2: 2 million cells) or combination NK+mAb9.2.27 with corresponding amounts of NK cells. In each cohort, 3–4 animals were included in the treatment groups.

### NK cell infusion and convection enhanced delivery of mAb 9.2.27

1 or 2 million NK cells were suspended in 10 µl of PBS and infused into the same coordinates as the tumour using a stereotactic frame prior to infusion of mAb9.2.27. A glass syringe (model 701, Hamilton, Bonaduz, Switzerland) secured in a microprocessor-controlled infusion pump (UMP 2–1, World Precision Instruments, Aston, Stevenage, UK) slowly delivered the NK cells over 5 minutes. After infusion, the needle was left in place for approximately 3 min and thereafter slowly retracted and the skinfolds closed with polyamide surgical thread (monotherapy NK cells). Otherwise, azide free mAb9.2.27 antibodies (4 mg/ml in 24% polyethylene glycol) were thereafter administered through a 26-gauge cannula connected to an osmotic mini pump (Mini-Osmotic pump model 2001D,Alzet Inc., Mountainview, CA)[16]. The pumps were installed while the animal was still in the stereotaxic frame, into the same coordinates as the tumour and NK cells and infused continuously over 24 h by convection enhanced delivery at a rate of 8 µl/h. The mAb9.2.27 was a generous gift from Professor Reisfeld (Scripps Institute for Cancer research, La Jolla, San Diego, CA, USA).

### MR imaging

Pre-treatment T1-weighted and T2-weighted scans were performed 3 weeks after tumour implantation to verify equal tumour load in the different groups. Post-treatment multiparametric MRI was performed at two time points; 7±2 and 17±1 days after treatment on a 7T Bruker Pharmascan (Bruker Biospin, Ettlingen, Germany), using a 38 mm rat head transmit and receive volume coil. The rats were anesthetized with 1–2% isoflurane in 1∶2 O_2_/N_2_, and positioned prone in a dedicated animal bed heated with recirculating warm water to keep the rat's body temperature stable at 37°C. Respiration and body temperature was monitored throughout the scanning session using BioTrig Life Monitoring System (Model 1025 Small Animal Monitoring and Gating System, SA Instruments). The multiparametric MR imaging protocol included the following scans: (1) T2-weighted RARE (rapid acquisition relaxation enhancement) sequence with repetition time (TR)  = 2400 ms, echo time (TE)  = 40 ms, rare factor  = 8, field of view (FOV)  = 35×35 mm^2^, matrix size  = 256×256, resolution  = 137×137 µm^2^/pixel, slice thickness of 1.5 mm (eight slices, no gap) and 12 averages, (2) diffusion weighted EPI (echo planar imaging) sequence with TR = 1500 ms, TE = 35 ms, Δ = 15 ms, δ = 7 ms, b-values in the range of 300–1200 s/mm^2^ (300, 500, 800 and 1200 s/mm^2^) and three orthogonal gradient diffusion directions, FOV = 35×35 mm^2^, matrix size  = 128×128, resolution  = 273×273 µm^2^/pixel, slice thickness of 1.5 mm (eight slices, no gap) and 10 averages; (3) RARE T1-map with TR = 125, 250, 500, 1200, 2500, 5500 and 10 000 ms, TE = 12.5 ms, rare factor  = 2, FOV = 35×35 mm^2^, matrix size  = 128×128, resolution  = 273×273 µm^2^/pixel, a slice thickness of 1.5 mm (four slices, no gap), (4) RARE T1-weighted pre-contrast with TR = 1300 ms, TE = 8.86 ms, rare factor  = 4, FOV = 35×35 mm^2^, matrix size  = 256×256, resolution  = 137×137 µm^2^/pixel, a slice thickness of 1.5 mm (eight slices, no gap) and 6 averages; (5) RARE T1-weighted dynamic contrast enhanced (DCE) series with 206 repetitions, TR = 200 ms, TE = 8.86 ms, rare factor  = 4, FOV = 35×35 mm^2^, matrix size  = 128×128, resolution  = 273×273 µm^2^/pixel and a slice thickness of 1.5 mm (four slices, no gap), scan time 16 min (temporal resolution  = 4.8s). In the dynamic scan, 0.2 mmol/kg Omniscan (gadodiamide, MW 0.58 kDa, GE Healthcare, Norway) was injected through the tail vein after acquisition of 20 baseline images. Another dose of gadodiamide was injected after the dynamic scan and prior to the acquisition of RARE T1-weighted post-contrast images with imaging parameters as described for the RARE T1-weighted pre-contrast sequence above. The total scan time was approximately 90 min including pilot scans. An additional follow-up MRI was performed on surviving animals 3 months after treatment start (RARE T2- and T1-weighted pre- and post-contrast images only).

### MR data analysis and quantification

MR data analysis was performed using in-house programs written in Matlab7.8.0 (The Mathworks, Inc, MA, USA). The data analysis included only data from animals with successful MRI scans (N = 3 in NK+mAb9.2.27 group, N = 5 or 6 NK cells only, N = 5 or 6 in mAb9.2.27 only and N = 5 or 6 in controls respectively for 7 and 17 days post-treatment). At 3 months, only structural MRI was performed on N = 3 surviving animals in cohort 2 in the NK+mAb9.2.27 combination therapy group. MRI data were excluded if unsuccessful contrast agent injections or pronounced movement artefacts were noted. Tumour size was measured in post-contrast T1-weighted images by manually drawing regions of interest (ROIs) of the entire volume of the tumour based on the signal intensity changes after contrast agent administration. By implementing a two-compartment model as described by Tofts [24], and using the T1-map from each individual animal, as well as the arterial input function measured by McGrath in rats using a contrast agent with the similar kinetic properties as gadodiamide [3], the transfer constant (K^trans^), extracellular extravascular volume fraction (v_e_) and blood plasma volume fraction (v_p_) were estimated. We also estimated the area under the curve at 1 min (AUC_1min_), which is a model independent semi-quantitative measure that reflects contrast agent kinetics determined by a combination of blood flow, volume and permeability [24]. All parameters were calculated on a voxel-by-voxel basis within the tumour volume. ADC maps (apparent diffusion coefficient) were calculated on a voxel-by-voxel basis from the diffusion weighted images with different b-values and in different directions as previously described [30] [31].

### Histological examination immunohistochemistry and quantification

Brains were formalin fixed, paraffin embedded, and every 20th 3–5 µm thick tissue section was collected for further histological analysis. These sections were stained with haematoxylin and eosin, and examined under a light microscope. Representative formalin-fixed, paraffin-embedded sections from each specimen were deparaffinised, epitope unmasked using the appropriate method for each antibody and immunohistochemically stained with various antibodies; anti-MIB-1 (Ki67, 1∶100, Dako, Glostrup, Denmark), using the avidin-biotin-peroxidase complex (Vectastain, Vector Laboratories, Burlingame, CA) method with 3, 3″-Diaminobenzidine (DAB) and H_2_O_2_ (DCS, Hamburg, Germany). The fraction of Ki67 positive against Ki67 negative tumour cells (Ki67 labelling index) was quantified in five manually and randomly selected hot spots in microscopic high power fields (400× magnification) in all animals in the study group using the NIS-Elements™ BR 3.1 software, (Nikon Corporation, Tokyo, Japan). Detection of apoptotic cells was performed with the terminal deoxynucleotide nick end-labelling (TUNEL) assay according to the manufacturer's instructions (Roche Applied Bioscience, Mannheim Germany). TUNEL-positive cells were visualised with DAB as chromogen, and the signal intensity of TUNEL-positive cells was assessed from the whole tumour section in 100× magnification in all the animals. The threshold level for pixel intensity was determined on the basis of four TUNEL stained tumour sections using a Nikon Eclipse 600 microscope. This threshold level was stored and subsequently applied with identical microscope settings for scoring of all tumour sections. Immune positive elements (pixels above threshold) were measured and expressed as area fraction of the visual field for each tumour section. The Growth Index (GI) was determined as the mean ratio of proliferative to apoptotic cells for all tumours in the groups.

### Statistical analysis

Immunohistochemistry data for the treatment groups was analysed using One way analysis of variance (ANOVA) with Bonferroni post hoc analysis correction for multiple testing in GraphPad Prism Statistical Software, version 5.0 (GraphPad, La Jolla, CA, USA). MRI derived parameters (K^trans^, v_e_, v_p_, AUC_1 min_, ADC and tumour size) were analysed in Stata v13.1 (StataCorp LP, Texas,USA) using linear models with robust variance estimates adjusted for clustering of rats. Post hoc analysis and correction for multiple testing was performed using Scheffe's adjustment based on the linear model, using the svy-module in Stata for the analysis. The figures were also based on the marginal means from the linear model clustered by rat. Statistical analyses were performed separately on the two cohorts and by combining the two first time points. Ordinary linear regression was performed to test for any associations between the MRI derived parameters. P-values less than 0.05 were considered statistically significant.

## Results

### Expression of glial markers and NK cell ligands on U87MG cells

**Figure 1:**
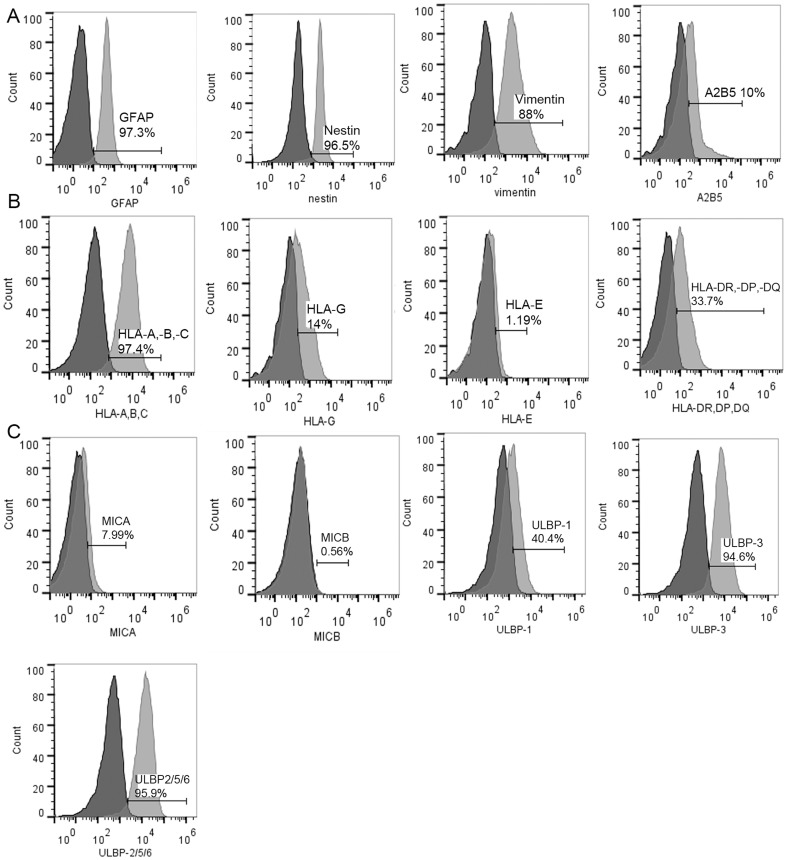
Expression of glial cell markers and NK cell ligands on U87MG tumour cells. (**A**) Mean Fluorescence Intensity (MFI) histograms and % cells expressing glial markers (GFAP,Nestin, Vimentin, and A2B5), **(B)** MFI histograms and % cells expressing class I HLA ligands (HLA-A,-B,-C), non-classical HLA-G and HLA-E, as well as HLA-DR,DP,DQ. **(C)** MFI histograms and % cells expressing ULBP 1, 3, 2/5/6, MICA and MICB activating ligands. Dark histograms represent negative control, light histograms represent stained cells.

We characterised the U87MG cells for markers of glial cells and demonstrated that 97.3% dimly expressed glial fibrilliary acidic protein (GFAP), 96.5% expressed nestin, 88% were vimentin positive and 10% expressed A2B5 (Figure 1 and Table 1). Previously we demonstrated that U87MG cells highly express NG2/CSPG4 [16]. To investigate whether U87MG cells also express ligands recognised by NK cell receptors, we characterised expression of class I and non-classical HLA molecules that are ligands for inhibitory KIRs and demonstrated that nearly all U87MG cells highly expressed HLA-A, -B,-C (97.4%). Fewer proportion of cells expressed HLA-G (14%), HLA-DR, DP,DQ (33.7%), and HLA-E (1.9%), Figure 1 and Table 1). The UL16 binding proteins (ULBPs) 1, 3, 2/5/6 were densely expressed on the majority of U87MG cells (40.4%, 94.6% and 95.9%, respectively, Figure 1C and Table 1). In contrast U87MG expressed low to negligible levels of MICA (7.99%) and MICB (0.59%) (Figure 1 and Table 1). MICA, MICB and the ULBPs are all stress induced ligands recognised by NKG2D activating NK cell receptor (Figure 1 and Table 1). Collectively, these data indicate that U87MG might be resistant to NK cell lysis.

**Table 1:** U87MG tumour cells' expression of glial markers and ligands for NK cell receptors.

Marker	% cells	MFI
HLA-ABC	97,4	6049
HLA-DR,DP,DQ	33,7	172
HLA-E	1,19	377
HLA-G	14	11,3
MICA	7,99	99,1
MICB	0	-
ULBP-1	40,4	2721
ULBP-2-5-6	95,9	12979
ULBP-3	94,6	7421
A2B5	10	637
Nestin	96,5	2208
Vimentin	88	2154
GFAP	97,3	397

### Morphological tumour progression

**Figure 2:**
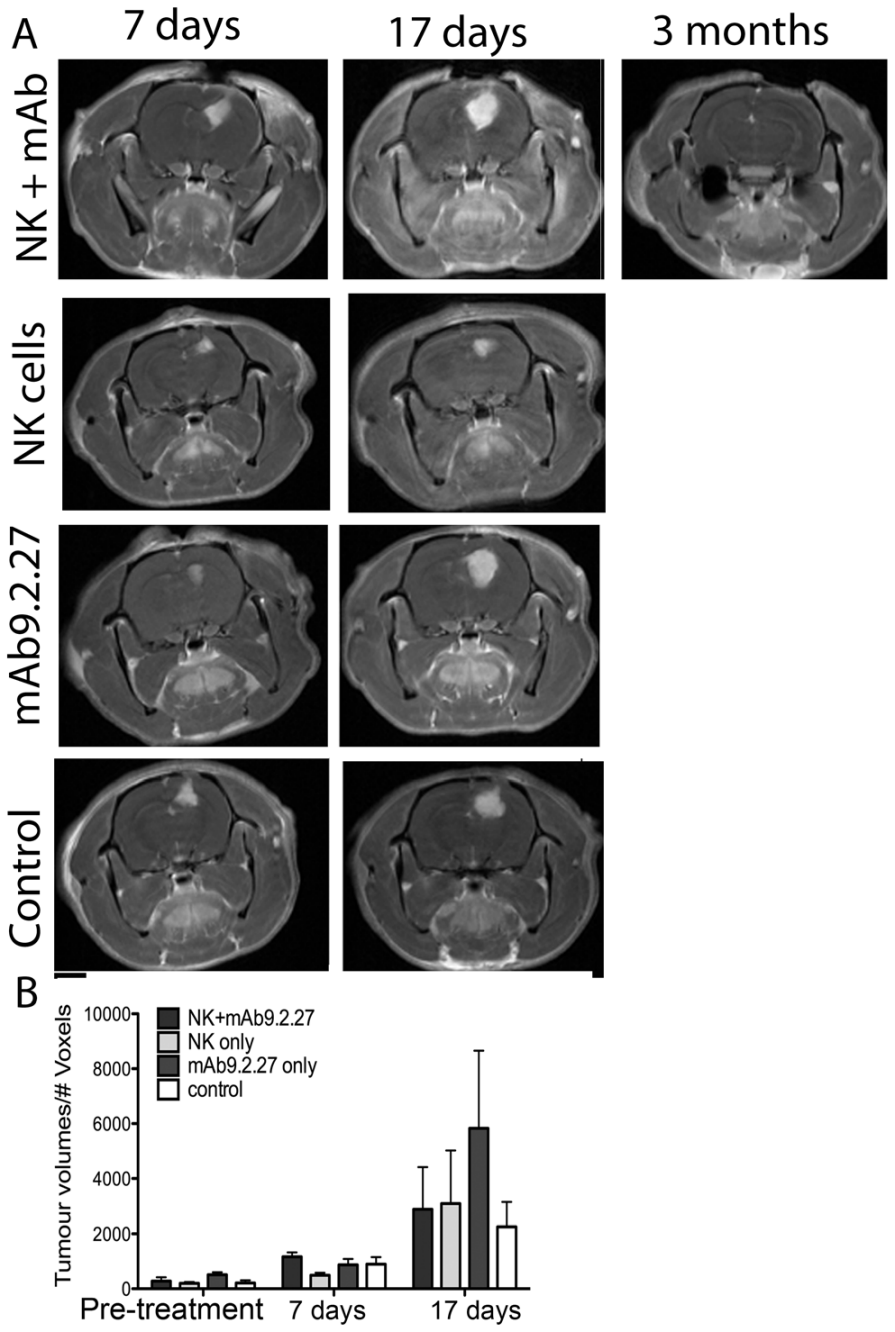
Longitudinal T1-weighted images and tumour growth. (**A**) Longitudinal axial post-contrast T1-weighted images of nude rats bearing U87MG tumours treated with combination NK+mAb9.2.27, NK cell monotherapy, mAb9.2.27 monotherapy, and vehicle controls, showing the same animal after 7 days, 17 days and 3 months post NK+mAb9.2.27 treatment. (**B**) Tumour volumes (#voxels) quantified on post-contrast T1-weighted images, before and after 7 and 17 days treatment. Data represents mean ±SEM of all animals treated.

**Figure 3:**
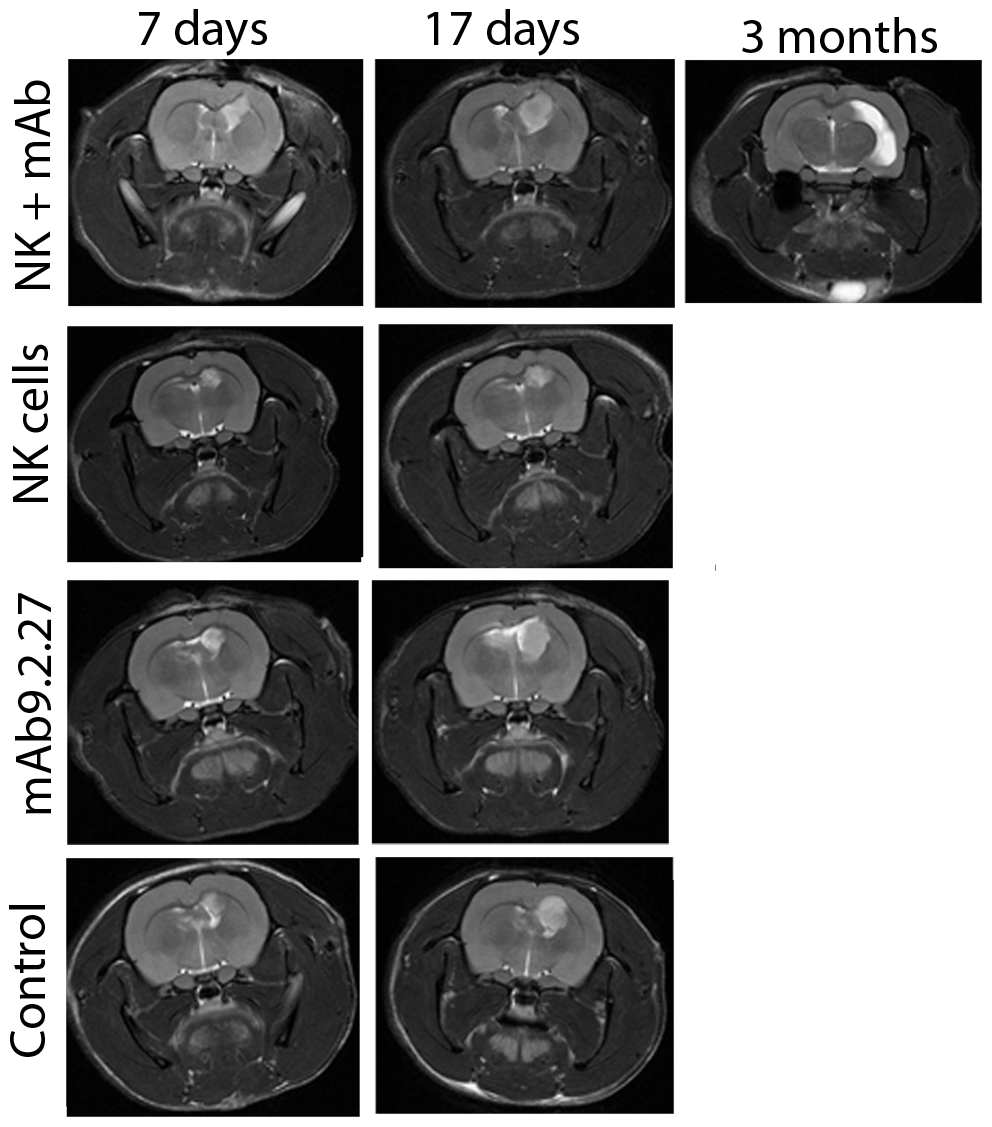
Longitudinal T2-weighted images. Longitudinal corresponding axial T2-weighted images of same animals as in Figure 2. Nude rats bearing U87MG tumours treated with combination NK+mAb9.2.27, NK cell monotherapy, mAb9.2.27 monotherapy, and vehicle controls, showing the same animal after 7 days, 17 days and 3 months post NK+mAb9.2.27 treatment.

**Figure 4:**
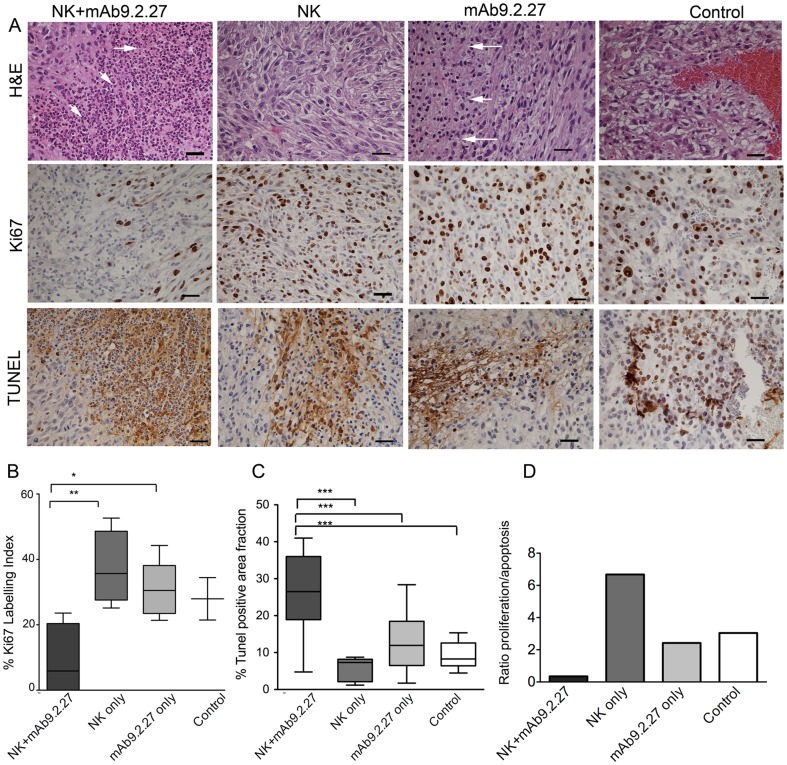
Immunohistochemical staining and cell proliferation. (**A**, top panel) haematoxylin and eosin staining showing leucocyte packed necrosis in U87MG tumours treated with NK+mAb9.2.27 and mAb9.2.27 monotherapy, (arrows), Magnification 200X; Scale bar 100 µm. Cellular dense tumours treated with NK cell monotherapy and haemorrhaging control, untreated tumours. (**A**, middle panel) Ki67 staining of proliferating tumour cells (**A**, bottom panel). Magnification 200X; scale bar 100 µm. Tunel stained apoptotic cells, Magnification 200X; Scale bar 100 µm. (**B**) Quantified Ki67 labelling index, data represents mean ±SEM of all animals treated. (**C**) Quantified Tunel labelling index, data represents mean ±SEM of all animals treated,*p<0.05; **p<0.001, ***p<0.0001. D Ratio of proliferation: apoptosis index.

T1-weighted post-contrast MR images of rats bearing U87MG tumours demonstrated contrast enhancement within the tumour mass in all tumours one week prior to treatment, indicating high angiogenic capacity and tumour progression (data not shown). Post-treatment images at 7±1 and 17±2 days revealed increased tumour volume in all groups in both cohorts, but with a large intra-group heterogeneity (Figure 2A and Figure 2B). No significant differences in mean tumour size between treatment groups was found in any of the cohorts (Figure 2B), Two-way ANOVA, p>0.05. Follow-up MRI scans performed on the NK+mAb9.2.27 treated animals that survived 3 months after treatment demonstrated reduction in tumour volume, where the animals did not have contrast enhancing tumour tissue left (Figure 2A). The affected areas were detected on T2-weighted images as oedematous regions with high signal intensity (Figure 3). Histological analyses at end-stage showed increased necrosis in the NK+mAb9.2.27 treated tumours compared to the mAb9.2.27 and NK cell monotherapy, as well as control untreated tumours, (Figure 4A). Tumour cell proliferation was diminished in the NK+mAb9.2.27 treated tumours (F = 7.4, p = 0.0039) compared to monotherapy NK cells (t = 4.29, p<0.001) and stand-alone mAb9.2.27 (t = 3.51, p<0.01), Figure 4A and 4B) with post-hoc Bonferroni adjusted analyses, after the ANOVA. For apoptosis the Bonferroni adjusted p-value showed that the NK+mAb9.2.27 combination treatment significantly promoted tumour cell death (F = 20, p = 0.0001), Figure 4A and 4C, compared to NK monotherapy (t = 5.22, p<0.0001), mAb9.2.27 only (t = 6.139, p<p<0.0001) and untreated controls (t = 5.20, p<0.0001). The growth index, estimated as the ratio of cell proliferation over apoptosis, demonstrated reduced tumour growth in the NK+mAb9.2.27 combination treatment group compared to the other groups (Figure 4D). The NK monotherapy group exhibited the highest growth index compared to the other groups (Figure 4D). Also, we found greater tumour infiltration by microglia/macrophages with pro-inflammatory phenotypes at end-stage in combination therapy group compared to monotherapy and control groups (Figure S1). Consequently, combination NK+mAb9.2.27 treatment significantly prolonged animal survival compared to NK cells and mAb9.2.27 monotherapy as we recently reported [16].

### Increased volume fraction in NK+mAb9.2.27 treatment responsive tumours

**Figure 5:**
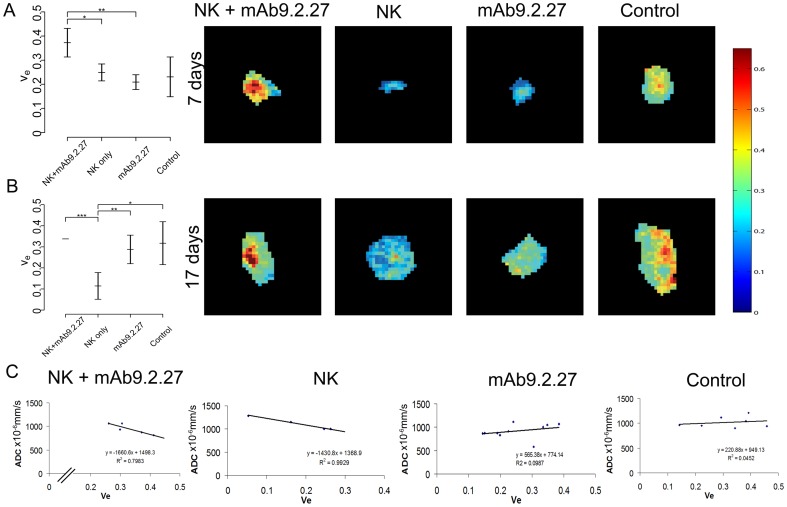
Perfusion parameters and maps. (**A**) Elevated extravascular extracellular volume fraction v_e_, in NK+mAb9.2.27 compared to monotherapy animals from cohort 1 (received 1 million NK cells), *p<0.05, **p<0.001 ***p<0.0001. (**A left panels**) Parametric maps visualising intratumoral heterogeneity in v_e_ in representative control, monotherapy and NK+mAb9.2.27 treated animals at 7 days. Intensity scale shows minimum (blue voxels) and maximum (red voxels) intensity levels. (**B**) Increased interstitial extracellular volume fraction, v_e_, elevated in NK+mAb9.2.27 compared to NK cells and mAb9.2.27 monotherapy animals, and decreased v_e_ in NK cell monotherapy compared to untreated control from cohort 2 (treated with 2 million NK cells), *p<0.05, **p<0.001, ***p<0.0001. (**B, left panels**) Parametric maps visualising intratumoural heterogeneity in v_e_ in control, monotherapy and NK+mAb9.2.27 treated animals at 17 days. (**C**) Significant association between ADC and v_e_ in the NK+mAb9.2.27 (R^2^ = 0.798, p = 0.041) and NK cell monotherapy animals (R^2^ = 0.993, p = 0.004). Graphs in A and B represent estimated marginal mean±95% confidence intervals.

DCE MRI detected changes in the tumour physiology earlier than the structural changes detected with conventional MRI. The parameter denoting extravascular extracellular volume fraction, v_e_, was significantly increased in the NK+mAb9.2.27 combination treated animals compared to NK cells monotherapy (Scheffe, t = −3.90, p = 0.017, CI −0.226 to −0.021) and mAb9.2.27 monotherapy (Scheffe, t = 5.32, p = 0.002, CI 0.064 to 0.263) in cohort 1 animals that were treated with 1 million NK cells, indicating reduction in cellular burden induced by combination treatment (Figure 5A). There was a small but not significant tendency to increased v_e_ in NK+mAb9.2.27 compared to untreated control (Scheffe, t = −3.04, p = 0.068, CI −0.293 to 0.08). These effects were reproducible in treatments with 2 million NK cells, where NK+mAb9.2.27 combination treatment significantly increased v_e_ compared to NK cells monotherapy (Scheffe, t = −7.97, p<0.0001, CI −0.304 to −0.142). NK cell monotherapy treatment decreased v_e_ compared to mAb9.2.29 (Scheffe, t = −4.22, p = 0.007, CI −0.293 to −0.534) and compared to untreated controls (Scheffe, t = 3.83, p = 0.013, CI 0.482 to 0.357) (Figure 5B). The difference in v_e_ between NK+mAb9.2.27 and mAb9.2.27 monotherapy was lost after increased dose of NK cells (Figure 5B). Parametric maps of v_e_ provided spatial information about treatment effects on a voxel-by-voxel basis (see Figure 5A and 5B), visualizing large intra-tumoural heterogeneity in the interstitial volume fraction in the NK+mAb9.2.27 combination therapy and control groups.

### Estimated parameters for blood flow, permeability and angiogenesis were not significantly affected by treatment

MRI derived parameters reflecting the vessel function and vessel wall integrity revealed less treatment dependent changes than the parameter denoting the interstitial volume fraction. The transfer constant K^trans^ describes the flux from vessels to interstitium but no effect of the treatment was found on K^trans^ in any of the cohorts. Furthermore, the model-independent parameter AUC_1 min_ was not significantly changed in any of the groups at any time point (data not shown). Also, the parameter reflecting the blood plasma fraction, v_p_, did not change significantly between groups. Correspondingly, at end-stage histology, there was no significant difference in the angiogenic capacity of the tumours (data not shown).

### Increased water diffusion associates with reduced volume fraction in non-responsive animals

Diffusion properties of water molecules in tumour interstitium was measured by DWI and expressed as ADC, which was increased in all tumours compared to normal brain tissue, most likely induced by perivascular oedema. However, mean ADC did not change significantly over time in any group and was not significantly different between the groups either. When performing correlation analysis of ADC values with DCE derived parameters, we could not find any overall association between ADC and K^trans^, v_e_ or AUC_1 min_. However, for animals treated with the NK+mAb9.2.27 combination treatment and NK cell monotherapy, there was a significant association between ADC and v_e_ (R^2^ = 0.798, p = 0.041 and R^2^ = 0.993, p = 0.004, Figure 5C). Interestingly, for both groups, an increased ADC value was associated with a reduction in v_e_. No associations were observed between ADC and extracellular volume fraction in control animals and animals treated with mAb9.2.27 alone.

## Discussion

The strategy of targeting the NG2/*CSPG4* proteoglycan with monoclonal antibodies in combination with the ADCC killing and cytokine modulating effect of NK cells proved to have the greatest therapeutic effect, compared to mAb9.2.27 or NK cell monotherapies. Therapeutic efficacy was validated at earlier time points by physiological MRI, and corroborated by histology and structural MRI findings at later stages. Four out of seven animals (60%) in the NK+mAb9.2.27 combination therapy group were alive at 3 months follow-up scans, whereas none were alive from the other treatment groups [16]. The infused rat NK cells expressed CD16 [16], a low affinity Fcγ-III receptor that binds antibody Fc domains to mediate activating signals that stimulate NK cells to kill coated target cells by ADCC. In our previous study we demonstrated that microglia in the tumour microenvironment were also able to bind the mAb9.2.27 via their Fc receptor to mediate effective GBM killing *ex vivo* [16]. These mechanisms are likely involved in the therapeutic efficacy of the combination treatment also in the present study. Nevertheless, we report herein that NK cell monotherapy failed to control GBM. The interstitial volume fraction, denoted by the MRI parameter v_e_, indicated that NK cell monotherapy failed to reduce the tumour cellular burden in the tumour microenvironment and this was corroborated by histological measures of decreased apoptosis, increased cell proliferation and subsequently, the poor survival outcomes of these animals [16]. This might be due to U87MG tumour cells' expression of elevated levels of class I HLA and non-classical HLA ligands for inhibitory receptors that supress NK cell activation against the tumour cells, as well as diminished expression of stress induced ligands for activating NK cell receptors. Although the HLA gene locus is highly pleomorphic with regard to gene content and allelic polymorphisms, the classical class I regions in humans, mice, and rats are relatively conserved. Nude rats express Ly-49 molecules [32] that recognize RT1-A, rodent orthologues to killer immunoglobulin like receptors (KIRs) and class I HLA (respectively), in humans. Given the low alloreactivity of the rat NK cells to the human GBM cells, we interpret that dominant inhibitory signals might have been transduced when rodent Ly-49 recognized the class I HLA ligands on U87MG cells, in addition to the non-classical HLA-G and HLA-E, in the absence of NKG2D dependent activation signals.

T1-weighted post-contrast images at 3 months and histological analyses revealed a regression of the NK+mAb9.2.27 combination treated tumours. Lack of contrast enhancement at the tumour region at this time point demonstrated treatment efficacy as confirmed by the diminished tumour tissue on histological staining. In our separate study, survival analyses demonstrated a correlation of NK+mAb9.2.27 treatment with prolonged survival in several tumour models [16].

There is currently a pressing need for imaging biomarkers that provide information about the tumour phenotypes in response to immunotherapy when it comes to tumour cell density, cell death and tumour-stromal tissue architecture. Changes in physiological parameters that correlate with therapeutic efficacy may be detected earlier in the treatment process than gross morphological changes such as tumour size. The findings reported in this study supported this hypothesis. We observed larger tumour lesions on contrast enhanced T1-weighted structural MR images after immunotherapy despite prolonged survival. This phenomenon may reflect genuine tumour progression or alternatively, it may be due to recruitment of immune cells into the tumour from the peripheral circulation. Indeed we recorded greater tumour infiltration by microglia/macrophages with pro-inflammatory phenotypes after combination NK+mAb9.2.27 compared to monotherapy and control animals at early stages [16] as well as at end-stage in the present study. Since the combination NK+mAb9.2.27 treatment diminished tumour growth index and prolonged survival, the contradictory increase in contrast enhancement might represent a false progression due to inflammatory processes. The increased contrast enhancement might be caused by a pronounced local tissue reaction with an inflammatory component, edema, and abnormal vessel permeability causing increased leakage of the contrast agent, and is most likely similar to the *so-called* flare effect, previously reported following immunotherapy with intralesionally infused lymphocytes in patients with recurrent high grade astrocytomas [33]. The flare effect is characterized by apparent worsening of the lesion in MRI images shortly after immunotherapy exhibiting increased nodular enhancement, increased oedema, and mass effect that resolves by 3 months in patients. Interestingly, we observed at the 3-month follow-up that the increased enhancement had resolved, with no further tumour recurrence in the NK+mAb9.2.27 treated animals. The flare effect was also reported following gene therapy [34], convection enhancement delivery of cytokines [35] or after placement of the GliaSite radiation therapy system [36] to treat brain tumour patients. This validates our findings that following immunotherapy, longitudinal multiparametric MRI should be performed to delineate whether the apparent increase of tumour enhancement and region of interest is due to the flare effect or to real tumour progression. Several papers point out this limitation of the RANO or Macdonald's radiological response criteria for immunotherapy [37] [5] [4] that use the enhancing tumour area as the primary measure of response, without considering the patients' use of steroids and changes in their neurologic status [38]. Tumour enhancement can occur following a variety of non-tumoural processes such as inflammation, seizure activity, postsurgical changes, and radiation necrosis [39]–[42]. Although the established criteria have limitations, in addition to the particular difficulty of measuring the often irregular shape of diffusely infiltrative gliomas, they are nevertheless widely accepted.

Here we have demonstrated that longitudinal physiological MR imaging provided more sensitive quantitative measures that allowed us to characterize tumour progression longitudinally. The derived parameter v_e_, that describes the volume fraction of the interstitium, proved to change earlier in the treatment process than the tumour volume, and may provide important information about the treatment course when traditional measures such as tumour volume fail. Others have also suggested this parameter as a candidate biomarker for the EES [43]. The volume fraction increased after NK+mAb9.2.27 treatment compared to the monotherapy and no treatment groups, despite adding more NK cells into the lesion. The increased extracellular extravascular space was due to augmented tumour cell death and reduced cellular proliferation as indicated by the diminished growth index on histological measures. Increased v_e_ in the combination treated animals thus correlated with efficient killing effect. Animals treated with NK cell monotherapy exhibited the greatest growth index, and v_e_ was decreased. The reduction was more pronounced in cohort 2 treated animal that received 2 million NK cells, indicating a dose dependent response. This can most likely be attributed to the presence of NK cells, in addition to the highly cellular tumour mass contributing to a tightly packed tumour cell mass, indicated also by histology. Thus, the volume of the interstitial space was reduced. The killing efficacy of NK cells alone was not sufficient to counteract the increase in cell numbers in the solid tumour mass, leading to reduced volume of interstitial space. This illustrates that mAb9.2.27 bound to the NG2/CSPG4 positive tumour cells was required to trigger ADCC mediated cytotoxicity and facilitate the efficacy of NK cells in this GBM model. The reduced interstitial volume fraction in the NK cell monotherapy group was accompanied by a tendency of reduction in the transvascular transport (K^trans^) and initial contrast uptake (AUC_1 min_). However, mechanical forces within the solid tumour play a role. Proliferating tumour cells induce physical stress that compresses blood vessels and therefore reduce the blood flow. Also, as a result of vessel leakiness and lack of functional lymphatics in most solid tumours, interstitial fluid pressure is significantly elevated, which is more profound in the inferior of the tumour [44], [45], [46]. As convection is one of the driving mechanisms of transvascular fluid transport, high interstitial pressure will work against efflux of fluid from the vessels, which in our study was reflected as reduced K^trans^ and AUC_1 min_. The estimated K^trans^ may also be influenced by blood flow and not exclusively by permeability when using low-molecular weight contrast agents [24], [47], [48]. This assumption complicates the interpretation of this parameter, and may explain why K^trans^ did not give any indication of treatment course in any of the groups.

The increased extracellular volume fraction expressed as v_e_, in the control tumours may be due to *de novo* necrosis caused by reduced blood supply, followed by hypoxia and cell death in the central parts of the tumour [29], [49]. This corresponds well with the histological analyses and the parametric maps of v_e_ and similar to other studies, where increasing v_e_ with increasing brain tumour grade has been reported [50]. The lack of significance in the estimated plasma volume v_p_ might be due to the fact that we used a literature based arterial input function (AIF) as baseline for the analyses. Ideally, AIF should be individually measured since v_p_ is highly dependent on the chosen AIF. However, all animals received a dose of the contrast agent dependent on the individual body weight, and the same injection time and speed were used, so inter-individual differences in the AIF were kept at the minimum. Also, inflow effects and slice positioning may influence the measured individual AIF and interfere with the result. An alternative explanation in the lack of differences in the plasma volume, is that the mAb9.2.27 is highly specific for the human CSPG4 epitope. Thus mAb9.2.27 monotherapy did not exhibit anti-angiogenic effects against the rat NG2 expressing tumour vessels. Indeed, no difference in microvascular densities as a measure of angiogenesis was demonstrated on immunohistochemistry.

ADC represents the random diffusion of water molecules within tissues and has been shown to be inversely correlated with tumour cell density [51]. ADC has furthermore been linked to cell death following treatment [52]. In this study we could not detect statistically significant changes in the estimated ADC value over time as a response to treatment. This is most likely due to the impact of other determinants of water diffusivity within the complex microenvironment encountered in gliomas. The presence of necrosis is an important diagnostic criterion for GBM that distinguishes this aggressive tumour from the low-grade tumours. The necrotic tissue may manifest as mobile fatty acid lipid moieties that are not associated with membrane phospholipids. Necrotic cell debris and deposits of blood after local haemorrhage are all factors that hinder the free movement of water molecules in the EES [43]. The ADC value is a fraction of both intra-cellular and extra-cellular diffusion [30] and is affected by not only the cellular density but also tissue tortuosity, extracellular components and their organization [53]. Additionally, intracellular diffusion and membrane permeability impact free movement of water and thus ADC. The ADC value was not affected by tumour size per se but highly dependent on the altered microarchitecture and physiology that characterize a malignant tumour.

Consistent with this, we found a statistically significant negative association between the ADC value and v_e_ in the NK+mAb9.2.27 combination therapy and NK cell monotherapy group, where high v_e_ values corresponded with low ADC values. This indicates that the ADC value and v_e_ may reflect different aspects of the tumour microenvironment and the ADC value may be more complex than simply reflecting the volume of the interstitial space. Our findings are supported by others [54] and [43]. In patients with glioma, no correlation between ADC and v_e_ was found and the authors suggested that the interpretation of these parameters is over simplified [43]. A negative correlation of ADC and v_e_ in breast tumour patients was reported [54]. However, they found that the ADC value was a better predictor of tumour response of chemotherapy than v_e_ in these patients. The ADC value is not only dependent on the volume of the EES but also the composition of the EES. Tumours are composed of the malignant cell compartment and the “benign” stromal cell compartment that includes blood vessels, infiltrating inflammatory cells, extracellular matrix, and stromal fibroblasts. The precise composition of the tumour microenvironment varies depending on the tumour site and histology. The brain, in particular, consists of numerous specialized cell types such as glia, brain endothelial cells and a relative paucity of extracellular matrix components. Therefore, the biological interpretation of the estimated MR-based parameters may vary between tumours of different histological origin, thus the tumour specific and treatment dependent physiology must always be taken into consideration. Other groups also failed to find a correlation between cell density and ADC in gliomas [55]. This corroborates our lack of correlation between v_e_ and “tumour size” at these early time points (3 weeks post treatment). This may be attributed to the high intra-tumour heterogeneity of GBMs with areas of oedema and cyst formation, vascular proliferation and necrosis, that all influence the estimated ADC value in numerous ways [43]. Also, the b-values have an impact on the ADC, as higher b-values (above 1000 s/mm^2^) weight the intracellular part of the ADC value more than lower b-values. The magnitude, duration, and temporal duration of the diffusion gradients determine the b-value and influence the signal observed in the DW images [43]. Several studies have concluded with that higher b-values (b = 3000 s/mm^2^) were more useful in discriminating low-grade and high-grade gliomas [56], [57] and to also discriminate true progression from pseudoprogression in patients with GBM undergoing radiation therapy and concomitant temozolomide [58].

To our knowledge, we are the first to report longitudinal and simultaneous measurements of both ADC and v_e_ in GBMs in the same animals. Being aware of the limited number of animals included in the study, our results indicate that v_e_ might be a reliable parameter for detecting early treatment response of targeted immunotherapy in GBMs using combined NK +mAb9.2.27. The v_e_ parameter is often overseen and not reported in many studies but may prove to be a candidate for the interstitial volume as it is a direct measurement of the EES [24]. However, the robustness of this parameter remains to be interrogated when targeting other receptors with different antibodies, therapy combinations, or mode of drug administration. More data will be required to establish a predictive model for treatment response based on MR derived physiological parameters. Utilisation of histogram analyses may provide more detailed information about tumour heterogeneity in GBMs.

Although, standard morphological MR imaging has played a pivotal role in defining landmarks used to manage GBM clinically, physiological MR imaging may hold the potential to improve diagnosis, prognosis, and prediction for individual patients' outcome in the long term. We have demonstrated that multiparametric longitudinal imaging provided a more comprehensive portrait of the tumour's microenvironment. Each MR sequence added additional knowledge about the tumour's response to treatment and allowed studies of the inter-related physiological parameters, which is a prerequisite for individualised treatment. Targeted immunotherapy against the NG2/CSPG4 proteoglycan in combination with the killing and cytokine modulating effect of NK cells improved therapeutic effects as determined by MRI.

## Supporting Information

Figure S1 **Comparison of tumour infiltrating immune cells after various treatments.** (**A**) Cellular composites of resident lymphocytes (CD45highCD11b/c low); macrophage/microglia (CD45+CD11b/c+) in control, mAb9.2.27 only, NK cell only and NK+mAb9.2.27 treated representative animals. (**B**) NK+mAb9.2.27 treated animals had greatest proportions of microglia that were similar to those in normal brain. The microglia were ED1+CD8+ and expressed MHC-class II molecules indicating an activated capable of antigen presentation. (**C**) Double staining for CD8 (brown) and ED1 (red) in histological tissues showing tumour infiltrating ED1/CD8+ macrophage/microglia, greatest infiltration in NK+mAb9.2.27 treated tumours. Arrows in panel C indicate tumour region, Magnification 200X, scale bar 100 µm. Data corroborates flow cytometric findings presented in panels A and B.
(TIF)

Materials and Methods S1 **Supporting Materials and Methods.**
(DOCX)
