# Supplementary figures and images for: Dynamic Contrast Enhanced MRI Detects Early Response to Adoptive NK Cellular Immunotherapy Targeting the NG2 Proteoglycan in a Rat Model of Glioblastoma
Source: PLoS One. 2014 Sep 30;9(9):e108414. doi: 10.1371/journal.pone.0108414 (PMC4182474; doi:10.1371/journal.pone.0108414)

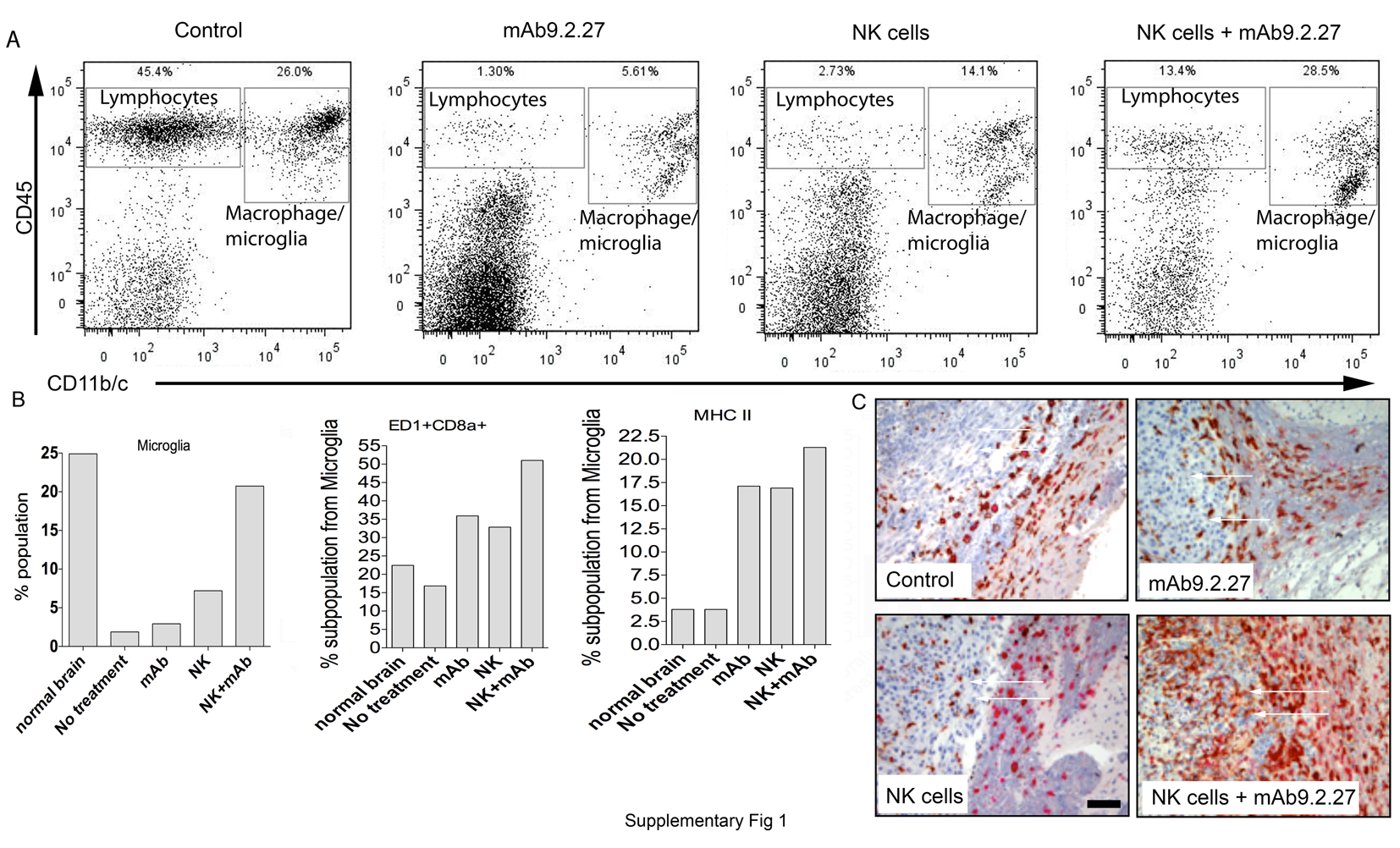

Supplement: Figure S1 — Comparison of tumour infiltrating immune cells after various treatments. (A) Cellular composites of resident lymphocytes (CD45highCD11b/c low); macrophage/microglia (CD45+CD11b/c+) in control, mAb9.2.27 only, NK cell only and NK+mAb9.2.27 treated representative animals. (B) NK+mAb9.2.27 treated animals had greatest proportions of microglia that were similar to those in normal brain. The microglia were ED1+CD8+ and expressed MHC-class II molecules indicating an activated capable of antigen presentation. (C) Double staining for CD8 (brown) and ED1 (red) in histological tissues showing tumour infiltrating ED1/CD8+ macrophage/microglia, greatest infiltration in NK+mAb9.2.27 treated tumours. Arrows in panel C indicate tumour region, Magnification 200X, scale bar 100 µm. Data corroborates flow cytometric findings presented in panels A and B. (TIF) [file pone.0108414.s001.tif]
