# Supplementary material for: Dynamic Contrast Enhanced MRI Detects Early Response to Adoptive NK Cellular Immunotherapy Targeting the NG2 Proteoglycan in a Rat Model of Glioblastoma
Source: PLoS One. 2014 Sep 30;9(9):e108414. doi: 10.1371/journal.pone.0108414 (PMC4182474; doi:10.1371/journal.pone.0108414)
Supplement: Materials and Methods S1 — Supporting Materials and Methods. (DOCX) [file pone.0108414.s002.docx]

**Supplementary Information**

**Supporting materials and methods S1**

*Flow cytometry*

The rat brain was dissociated into single cell suspension as previously reported (Svendsen et al., 2011). The rat brain cells were stained with APC-Cy7–conjugated anti-CD45 (OX-1, Biolegend, DS Uithoorn, The Netherlands), PE-Cy7-conjugated anti-CD8 (OX-8, eBioscience), PerCP-efluor 710-conjugated anti-CD11b/c (OX-42, eBioscience), or anti-MHC class II (HIS19, eBioscience). All samples were stained with LIVE/DEAD fixable Yellow (Invitrogen) in order to gate out dead cells. Then the cells were fixed and permeabilized using CytofixCytoperm solution (BD Biosciences) before staining with Alexa fluor 700-conjugated anti-CD68 (ED1, abD Serotec). Before data acquisition using FACS Fortessa (BD Biosciences, Erembodegem, Belgium), nucleated cells were stained using Sytox blue (Invitrogen). Data analysis was performed in FACSDiva Software version 6.1.2 (BD Biosciences) and figures prepared in Flowjo (Tree Star).
